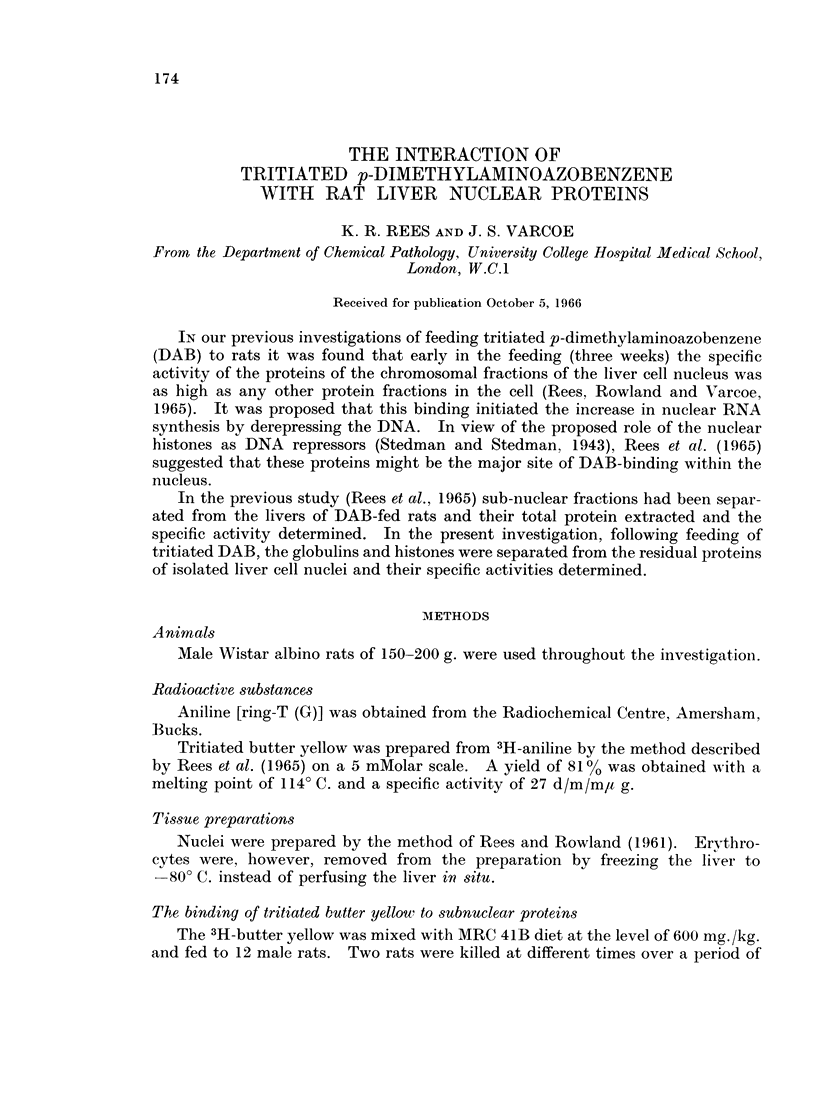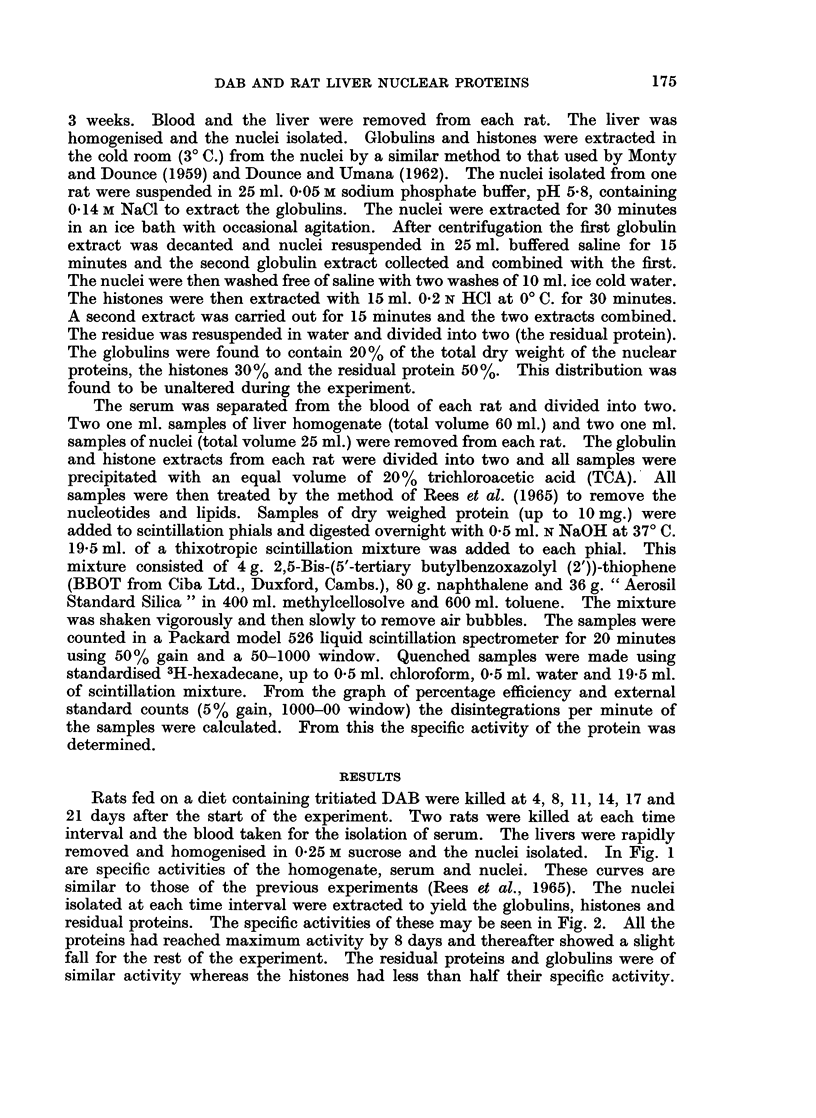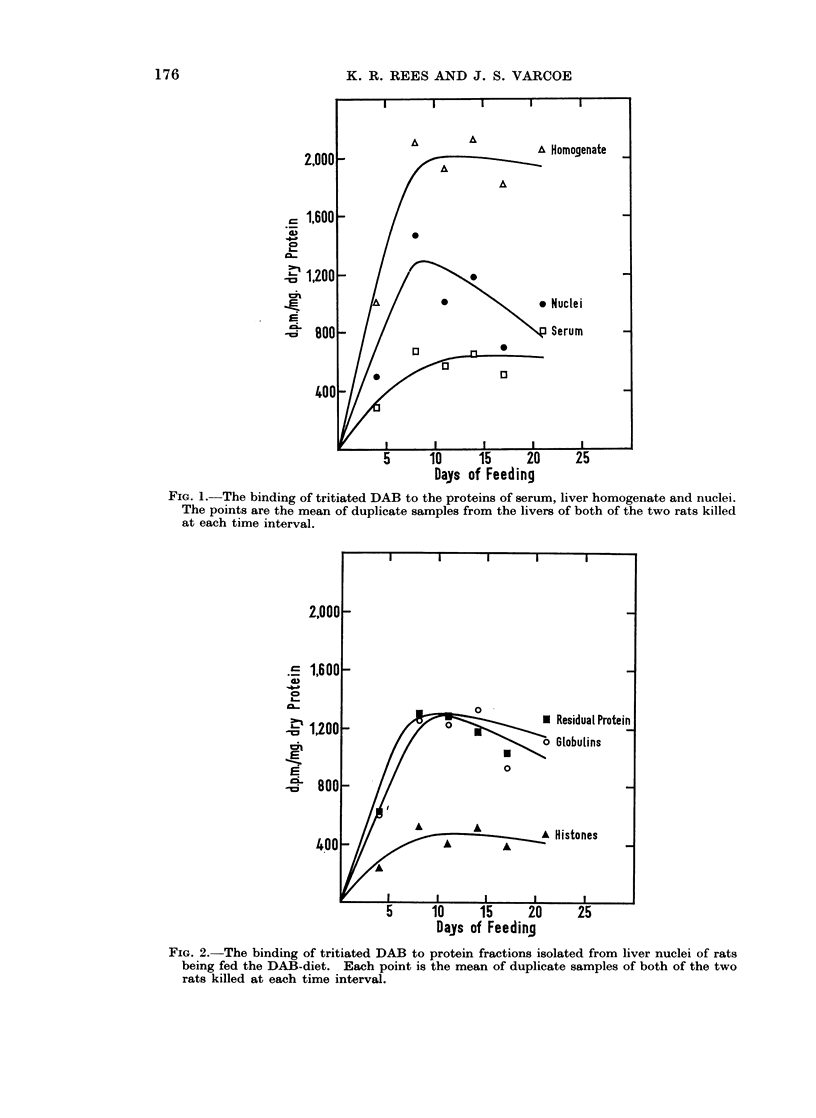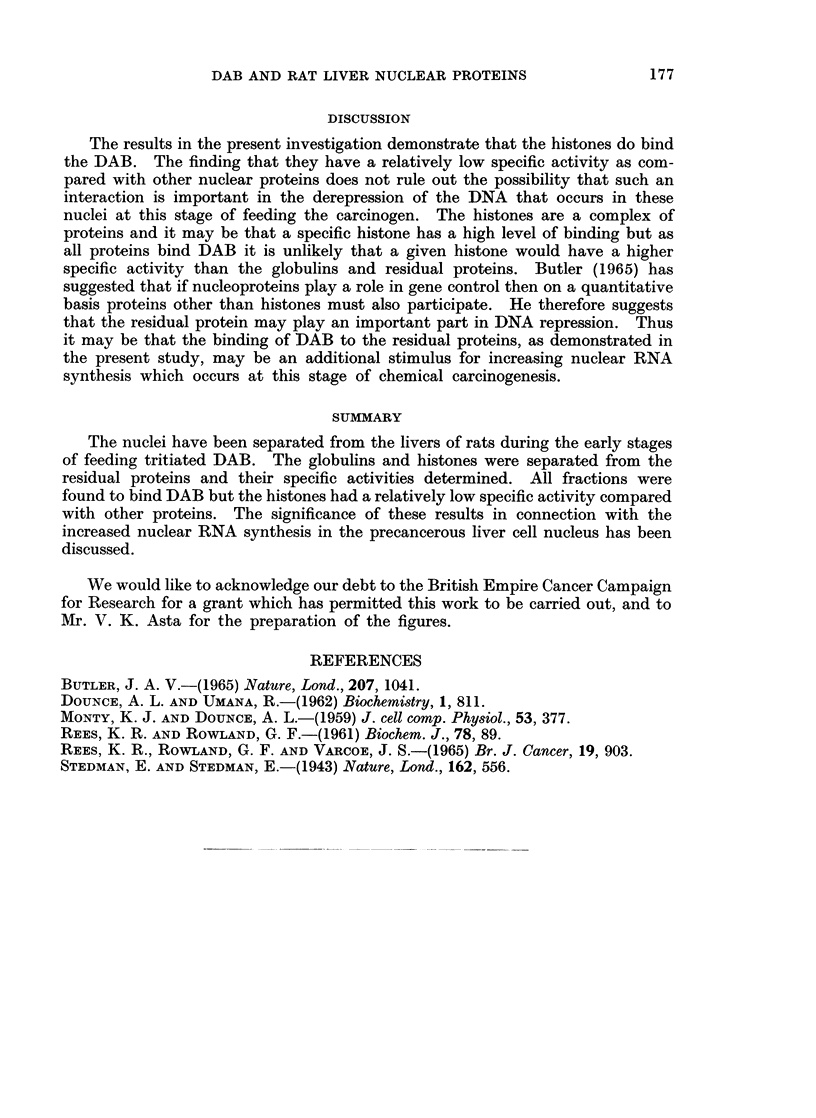# The interaction of tritiated rho-dimethylaminoazobenzene with rat liver nuclear proteins.

**DOI:** 10.1038/bjc.1967.17

**Published:** 1967-03

**Authors:** K. R. Rees, J. S. Varcoe


### THE INTERACTION OF TRITIATED *p*-DIMETHYLAMINOAZOBENZENE WITH RAT LIVER NUCLEAR PROTEINS

K. R. REES AND J. S. VARCOE

*From the Department of Chemical Pathology, University College Hospital Medical School, London, W.C.*1

Received for publication October 5, 1966

IN our previous investigations of feeding tritiated *p*-dimethylaminoazobenzene (DAB) to rats it was found that early in the feeding (three weeks) the specific activity of the proteins of the chromosomal fractions of the liver cell nucleus was as high as any other protein fractions in the cell (Rees, Rowland and Varcoe, 1965). It was proposed that this binding initiated the increase in nuclear RNA synthesis by derepressing the DNA. In view of the proposed role of the nuclear histones as DNA repressors (Stedman and Stedman, 1943), Rees *et al.* (1965) suggested that these proteins might be the major site of DAB-binding within the nucleus.

In the previous study (Rees *et al.*, 1965) sub-nuclear fractions had been separated from the livers of DAB-fed rats and their total protein extracted and the specific activity determined. In the present investigation, following feeding of tritiated DAB, the globulins and histones were separated from the residual proteins of isolated liver cell nuclei and their specific activities determined.

### METHODS

### *Animals*

Male Wistar albino rats of 150–200 g. were used throughout the investigation.

### *Radioactive substances*

Aniline [ring-T (G)] was obtained from the Radiochemical Centre, Amersham, Bucks.

Tritiated butter yellow was prepared from $^{3}H$-aniline by the method described by Rees *et al.* (1965) on a 5 mMolar scale. A yield of 81% was obtained with a melting point of 114° C. and a specific activity of 27 d/m/m$\mu$ g.

### *Tissue preparations*

Nuclei were prepared by the method of Rees and Rowland (1961). Erythrocytes were, however, removed from the preparation by freezing the liver to —80° C. instead of perfusing the liver *in situ.*

### *The binding of tritiated butter yellow to subnuclear proteins*

The $^{3}H$-butter yellow was mixed with MRC 41B diet at the level of 600 mg./kg. and fed to 12 male rats. Two rats were killed at different times over a period of

3 weeks. Blood and the liver were removed from each rat. The liver was homogenised and the nuclei isolated. Globulins and histones were extracted in the cold room (3° C.) from the nuclei by a similar method to that used by Monty and Dounce (1959) and Dounce and Umana (1962). The nuclei isolated from one rat were suspended in 25 ml. 0·05 M sodium phosphate buffer, pH 5·8, containing 0·14 M NaCl to extract the globulins. The nuclei were extracted for 30 minutes in an ice bath with occasional agitation. After centrifugation the first globulin extract was decanted and nuclei resuspended in 25 ml. buffered saline for 15 minutes and the second globulin extract collected and combined with the first. The nuclei were then washed free of saline with two washes of 10 ml. ice cold water. The histones were then extracted with 15 ml. 0·2 N HCl at 0° C. for 30 minutes. A second extract was carried out for 15 minutes and the two extracts combined. The residue was resuspended in water and divided into two (the residual protein). The globulins were found to contain 20% of the total dry weight of the nuclear proteins, the histones 30% and the residual protein 50%. This distribution was found to be unaltered during the experiment.

The serum was separated from the blood of each rat and divided into two. Two one ml. samples of liver homogenate (total volume 60 ml.) and two one ml. samples of nuclei (total volume 25 ml.) were removed from each rat. The globulin and histone extracts from each rat were divided into two and all samples were precipitated with an equal volume of 20% trichloroacetic acid (TCA). All samples were then treated by the method of Rees *et al.* (1965) to remove the nucleotides and lipids. Samples of dry weighed protein (up to 10 mg.) were added to scintillation phials and digested overnight with 0·5 ml. N NaOH at 37° C. 19·5 ml. of a thixotropic scintillation mixture was added to each phial. This mixture consisted of 4 g. 2,5-Bis-(5′-tertiary butylbenzoxazolyl (2′))-thiophene (BBOT from Ciba Ltd., Duxford, Cambs.), 80 g. naphthalene and 36 g. " Aerosil Standard Silica " in 400 ml. methylcellosolve and 600 ml. toluene. The mixture was shaken vigorously and then slowly to remove air bubbles. The samples were counted in a Packard model 526 liquid scintillation spectrometer for 20 minutes using 50% gain and a 50–1000 window. Quenched samples were made using standardised $^{3}$H-hexadecane, up to 0·5 ml. chloroform, 0·5 ml. water and 19·5 ml. of scintillation mixture. From the graph of percentage efficiency and external standard counts (5% gain, 1000–00 window) the disintegrations per minute of the samples were calculated. From this the specific activity of the protein was determined.

### RESULTS

Rats fed on a diet containing tritiated DAB were killed at 4, 8, 11, 14, 17 and 21 days after the start of the experiment. Two rats were killed at each time interval and the blood taken for the isolation of serum. The livers were rapidly removed and homogenised in 0·25 M sucrose and the nuclei isolated. In Fig. 1 are specific activities of the homogenate, serum and nuclei. These curves are similar to those of the previous experiments (Rees *et al.*, 1965). The nuclei isolated at each time interval were extracted to yield the globulins, histones and residual proteins. The specific activities of these may be seen in Fig. 2. All the proteins had reached maximum activity by 8 days and thereafter showed a slight fall for the rest of the experiment. The residual proteins and globulins were of similar activity whereas the histones had less than half their specific activity.

FIG. 1.—The binding of tritiated DAB to the proteins of serum, liver homogenate and nuclei. The points are the mean of duplicate samples from the livers of both of the two rats killed at each time interval.

FIG. 2.—The binding of tritiated DAB to protein fractions isolated from liver nuclei of rats being fed the DAB-diet. Each point is the mean of duplicate samples of both of the two rats killed at each time interval.

### DISCUSSION

The results in the present investigation demonstrate that the histones do bind the DAB. The finding that they have a relatively low specific activity as compared with other nuclear proteins does not rule out the possibility that such an interaction is important in the derepression of the DNA that occurs in these nuclei at this stage of feeding the carcinogen. The histones are a complex of proteins and it may be that a specific histone has a high level of binding but as all proteins bind DAB it is unlikely that a given histone would have a higher specific activity than the globulins and residual proteins. Butler (1965) has suggested that if nucleoproteins play a role in gene control then on a quantitative basis proteins other than histones must also participate. He therefore suggests that the residual protein may play an important part in DNA repression. Thus it may be that the binding of DAB to the residual proteins, as demonstrated in the present study, may be an additional stimulus for increasing nuclear RNA synthesis which occurs at this stage of chemical carcinogenesis.

### SUMMARY

The nuclei have been separated from the livers of rats during the early stages of feeding tritiated DAB. The globulins and histones were separated from the residual proteins and their specific activities determined. All fractions were found to bind DAB but the histones had a relatively low specific activity compared with other proteins. The significance of these results in connection with the increased nuclear RNA synthesis in the precancerous liver cell nucleus has been discussed.

We would like to acknowledge our debt to the British Empire Cancer Campaign for Research for a grant which has permitted this work to be carried out, and to Mr. V. K. Asta for the preparation of the figures.

### REFERENCES

BUTLER, J. A. V.—(1965) *Nature, Lond.*, **207**, 1041.
DOUNCE, A. L. AND UMANA, R.—(1962) *Biochemistry*, **1**, 811.
MONTY, K. J. AND DOUNCE, A. L.—(1959) *J. cell comp. Physiol.*, **53**, 377.
REES, K. R. AND ROWLAND, G. F.—(1961) *Biochem. J.*, **78**, 89.
REES, K. R., ROWLAND, G. F. AND VARCOE, J. S.—(1965) *Br. J. Cancer*, **19**, 903.
STEDMAN, E. AND STEDMAN, E.—(1943) *Nature, Lond.*, **162**, 556.